# Evaluation of the Effect of Selected Physiological Fluid Contaminants on the Mechanical Properties of Selected Medium-Viscosity PMMA Bone Cements

**DOI:** 10.3390/ma15062197

**Published:** 2022-03-16

**Authors:** Robert Karpiński, Jakub Szabelski, Przemysław Krakowski, Mariusz Jojczuk, Józef Jonak, Adam Nogalski

**Affiliations:** 1 Department of Machine Design and Mechatronics, Faculty of Mechanical Engineering, Lublin University of Technology, Nadbystrzycka 36, 20-618 Lublin, Poland; j.jonak@pollub.pl; 2 Section of Biomedical Engineering, Department of Computerization and Production Robotization, Faculty of Mechanical Engineering, Lublin University of Technology, Nadbystrzycka 36, 20-618 Lublin, Poland; 3 Department of Trauma Surgery and Emergency Medicine, Medical University of Lublin, Staszica 11, 20-081 Lublin, Poland; przemyslaw.krakowski84@gmail.com (P.K.); mariusz.jojczuk@umlub.pl (M.J.); adamnogalski5@gmail.com (A.N.); 4 Orthopaedic Department, Łęczna Hospital, Krasnystawska 52, 21-010 Leczna, Poland

**Keywords:** bone cement, contamination, blood, saline, mechanical parameters, compressive strength, modulus of elasticity

## Abstract

Revision surgeries several years after the implantation of the prosthesis are unfavorable from the patient’s point of view as they expose him to additional discomfort, to risk of complications and are expensive. One of the factors responsible for the aseptic loosening of the prosthesis is the gradual degradation of the cement material as a result of working under considerable loads, in an aggressive environment of the human body. Contaminants present in the surgical field may significantly affect the durability of the bone cement and, consequently, of the entire bone-cement-prosthesis system. The paper presents the results of an analysis of selected mechanical properties of two medium-viscosity bone cements DePuy CMW3 Gentamicin and Heraeus Palamed, for the samples contaminated with saline and blood in the range of 1–10%. The results obtained for compressive strength and modulus of elasticity were subjected to statistical analysis, which estimated the nature of changes in these parameters depending on the amount and type of contamination and their statistical significance.

## 1. Introduction

Osteoarthritis is a progressive, incurable disease that mostly affects the elderly; however, an increasing number of studies are showing osteoarthritic changes in the younger population [1,2,3,4]. Some factors such as obesity, injuries, and work and leisure joint overload can accelerate the development of osteoarthritis [5,6,7,8,9,10]. Hyaline cartilage is a highly sophisticated tissue, which is responsible for painless and smooth movement of joints. However, due to its highly specialized structure, low chondrocyte count and slow metabolism, its healing capacity is relatively low [11]. Cartilage, which cannot heal properly, will not perform its function, therefore pain and loss of movement in affected joints will occur as an effect of osteoarthritic changes. Gold standard for end stage disease is total joint replacement. In 1954, Shiers [12] published his paper on the use of metal implants in osteoarthritic knee, which started development of joint replacement surgeries on wider scale. In recent years, the number of total joint replacement procedures is increasing, and between 2003 and 2014, the increase reached 115.1% [13]. It is suggested that up to 2040, the increase in total joint replacement rate will be 400% [14]. Bone cements were firstly introduced by Charnley [15] and are one of the most commonly used polymer composites in in dentistry and orthopaedics surgery [16]. Apart from binding endoprosthesis with bone, they are also used for filling bone defects, strengthening bone in pathological fractures or during minimally invasive vertebroplasty [17,18,19,20]. According to Swedish Knee Arthroplasty Register majority of total knee replacements (TKR) are performed with the use of bone cement, and cementless TKR is susceptible to higher revision rate [21]. The 15-year survivor rate of total joint replacement is estimated to be about 90% [22]. Therefore, revision rate of total joint replacement may significantly increase in future years. Two main reasons for revision surgery are aseptic loosening and infection. It is estimated that 1–2% of all total joint replacements will develop fast or delayed infection [22]. The second most common cause of revision is aseptic loosening of the endoprosthesis. During daily activities, joint prostheses are subjected to great loads and transmit high forces. These loads are also transferred to the bone cement, which is the only connection between the bone and the endoprosthesis. This means that the strength of the bone cement is crucial for the survival of the endoprosthesis. Many factors influence bone cement properties, out of which fatigue, viscoelasticity of creep and stress relaxation are of paramount importance in cemented endoprosthesis [23].

In general, bone cements are self-polymerizing biomaterials that are widely used in orthopedic, traumatology and oncologic, spine or maxillofacial surgery when bone defects need to be filled [24,25]. Cementation technique has a significant impact on the survival of orthopaedic implants. Despite optimized cement preparation and proper pre-cementing technique, the biomechanical properties of the bone cement used appear to be very important in preventing aseptic loosening of the implant [26,27]. The mechanical properties of bone cements may be affected by factors such as antibiotic content; intentionally introduced admixtures [28,29,30,31,32]; or contaminants present in the surgical field such as blood, bone tissue fragments, saline solution [33,34,35,36] or commonly used lavage solutions [37], as well as the mixing process itself [38,39,40] and the preparation of the cement for implantation. Intensive research is currently being conducted to improve the thermal, mechanical and biological properties of bone cements [16]. The research includes doping the cement mass with small amounts of components such as carbon fibers [41], zirconia fibers [42], graphite fibers [43], graphene oxide [44,45,46,47], bioactive glasses [48], nanosilver [49], polydioxanone (PDO) [48], cellulose [48,50,51], mesoporous silica nanoparticles [52,53], aramid [54,55], polyethylene [56], titanium [57,58], ultra-high molecular weight polyethylene [59], tricalcium phosphate (TCP) [16,60] or hydroxyapatite (HA) [61,62]. The effect of aging processes associated with the absorption of physiological fluids and the accompanying hydrolysis of polymethylmethacrylate (PMMA) occurring in the outermost layers of the cement are also important, as well as the effect of deviation from the manufacturer’s recommended cement mix ratio [63,64,65,66,67]. It is important to remember that the post-implant cement works in the aggressive environment of the human body and is subjected to cyclical stresses. These are factors that determine the long-term survival of the bone-cement-prosthesis connection. [68]. Considering the fact that cement is the weakest element of the bone-cement-prosthesis system, it is extremely important to study the effects of factors that may worsen its mechanical properties immediately after implantation or accelerate the ageing process, allowing for the cement to lose its mechanical properties prematurely, which may result in prosthesis loosening [69]. In this paper, the authors present the results of studies on the influence of admixing the cement mass with impurities in the form of physiological fluids naturally occurring in the surgical field (blood and 0.9% saline solution) on the mechanical properties of selected commercially available bone cements. Such conditions can occur during TJR implantation, if the manufacturer’s guidelines will not be fulfilled correctly by the surgeon. Therefore, understanding the effect of bone cement contamination is not only a theoretical problem but a genuine issue for surgeons and patients.

## 2. Materials and Methods

### 2.1. Materials and Sample Preparations

The list of known bone cements includes more than 70 products from about 20 different manufacturers, of which about 50 types are still commercially available. They differ in application method, strength characteristics, time and temperature of curing and many other parameters [70,71]. Samples made of two commercial cements were tested: DePuy CMW3 Gentamicin (G) and Heraeus Palamed. The selection of cements was based on a combination of materials with similar properties. Palamed (Wehrheim, Germany) is medium-viscosity, fast-curing, radiopaque, poly-(methyl methacrylate)-based bone cement. To improve visibility in the surgical field, the cement has been coloured with chlorophyll (E141). The X-ray contrast medium is zirconium dioxide. The packaging contains gentamicin-containing polymer powder and a brown glass ampoule of liquid monomer, which are mixed cured in exothermic reaction for approximately 10 min depending on the temperature. DePuy CMW3 Gentamicin (Raynham, MA, USA) is composed of medium-viscosity, self-curing, radiopaque, polymethyl-methacrylate-based cements, containing antibiotics, and is used for securing a metal or polymeric prosthesis to living bone in arthroplasty procedures. It is primarily intended for syringe application, but if it is applied digitally, the surgeon must use their clinical judgement to decide when the cement is of a suitable viscosity to allow the surgical procedure to continue.

A summary of the chemical composition of both cements is shown in Table 1. The compositions of the analysed cements are similar. The most important dissimilarities are the use of different radiopaque agents and the presence of a colorant in Palamed (both in the liquid and powder part). In addition, an antibiotic was used in the CMW3 cement. Of course, the cements may differ in the amount of individual common components, which will affect the final strength characteristics of the individual cements.

**Figure 1:**
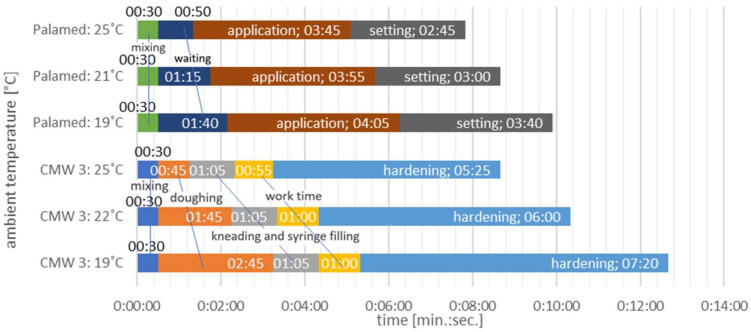
Working times for manual mixing (not pre-chilled bone cement).

The research was planned and carried out on the basis of ISO 5833 standard: implants for surgery—acrylic resin cements [72] and annex E: determination of compressive strength of polymerized cement. The precooled monomer and liquid part of the cement were mixed by hand at temperature of 20 °C for time of approx. 2 min, keeping in mind the working times for manual mixing of each cement [35] (Figure 1). The bone cement was mixed with physiological fluid. Physiological fluids are used during surgical field irrigation prior to cement placement; therefore, in surgical practice such contamination is unavoidable. In this study, we have used commercial 0.9 %NaCl solution, which is isotonic to blood in its nature and is commonly used for intravenous fluid infusions, wound cleansing and surgical wounds irrigation. Approval of Scientific Research Ethics Committee of the Lublin University of Technology was obtained with consent number KE-05/2016.

The blood samples were collected from volunteers after explanation of the procedure, explanation of possible side effects of blood collection and the signing of written consent by each volunteer. Prior to blood sample collection, the skin was cleansed with antiseptic solution and intravenous canula was introduced. Blood was sampled in a typical manner by intravenous canula introduced into cubital fossa vain. Blood samples were collected without any additives such as anticoagulants to reflect surgical field blood contamination prior to cement introduction. Blood samples were mixed with bone cement immediately after collection from volunteers and were not altered in any way. Blood collection was supervised by a health care professional with adequate certification. Any remaining biological and biohazard wastes were utilized according to appropriate regulations.

Contaminants were introduced into the bone cement at preparation stage. This approach was deliberately chosen to mix whole cement mass with the contaminant and not only the peripheral layers of specimen, and secondly to reduce variables such as time which could influence the results. It was shown that the longer bone cement is immersed in a contaminant solution, the higher absorption of contaminant that existed in the sample [63]. Based on those findings, full-contamination testing was chosen in this study. A specified weight quantity of contaminant was added to even amount of uncured cement and mixed. Contamination ratio is given in relative units % *w*/*w*. This approach enabled investigation of the contaminated cement strength impairment in extreme conditions, which, however, could happen in live surgery while the thickness of cement mass in intramedullary canal is relatively low.

**Figure 2:**
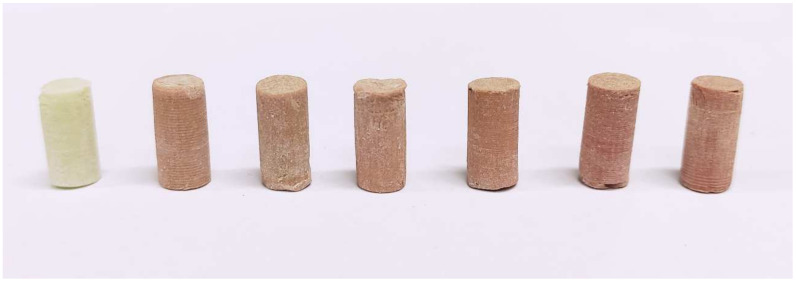
Samples in order of degree of blood contamination (Palamed).

The tests were carried out for different cases of quantitatively variable degree of cement mass contamination, in the range of 0–10% by weight. Using cast, cylinders samples were prepared, no less than 7 per combination of contaminant amount. The final dimensions of ⌀6 ± 1 mm × 12 ± 1 mm were obtained after mild abrasive treatment of both of the ends of the cement cylinders planes with the faces of the mould. The final samples contaminated with saline did not differ much in terms of colour. Those with added blood were darker after each step of contamination increase (Figure 2).

### 2.2. Mechanical Testing

**Figure 3:**
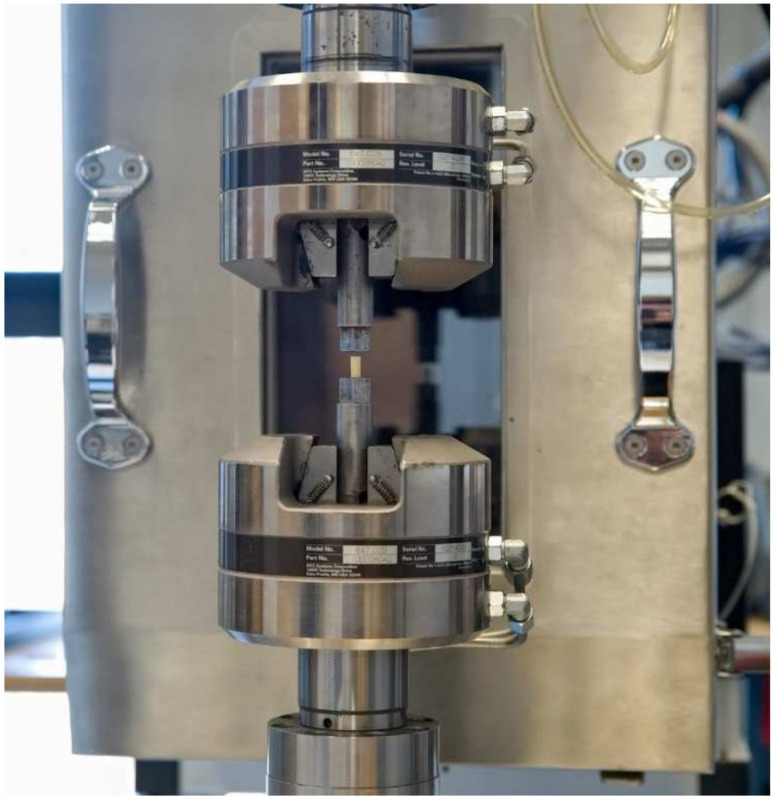
Testing machine grips with bone cement sample.

The compressive strength of the cylinders was determined using MTS Bionix–Servohydraulic Test System (Eden Prairie, MN, USA)—the test machine capable of applying and measuring a compressive force, equipped to record load versus crosshead displacement (Figure 3). The average diameter of each test piece was measured prior the test. The curves of displacement against load, using a constant cross-head speed of 20 mm/min, were obtained. Upper yield-point load divided by the original cross-sectional area of the cylinder was used to express the compressive strength. In addition, the stiffness of the material—compressive modulus of elasticity of examined cements—was calculated as the slope of the stress-strain curve at 2% displacement, in the area of the linear elastic strain region. Compression/compressive modulus is also known as compressive Young’s modulus and describes ability of the material to withstand changes in length when subjected to compressive loads. The higher the compression modulus, the stiffer the material. The examined specimens were tested at 23 °C.

### 2.3. Statistical Analysis

The test results, as recommended by ISO 5833, are presented as mean values and standard deviations. However, this is not sufficient information to draw conclusions about statistically significant differences between individual batches of samples. Therefore, statistical analyses were performed that, at a significance level of α = 0.05, will allow one to estimate the actual changes in compressive strength and compressive modulus of elasticity as a function of the amount of contamination. The tests were conducted using the software of TIBCO Software Inc. (2017) Statistica (data analysis software system), version 13.3.

The methods of multiple comparison of averages of several groups in order to clarify the differences detected by the analysis of variance allow for the grouping of the mean values and extract homogeneous groups, i.e., groups of mean values that do not differ statistically from each other. From the available solutions (Scheffé, Tukey, Newman and Keuls, Duncan, Fisher tests), the Tukey test was selected, namely, its variant for unequal samples, as the tested groups differed in the number of correct samples [73].

## 3. Results and Discussion

### 3.1. Compressive Strength

**Figure 4:**
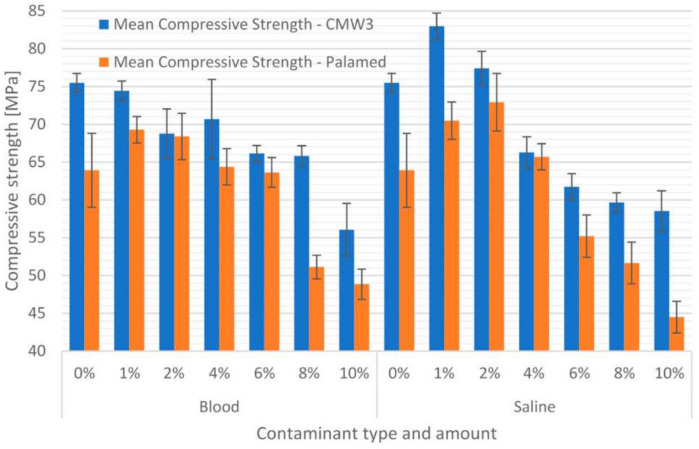
Compressive strength of contaminated bone cements.

The final results of the compressive test are presented in Figure 4 and Table 2. The relatively low values of the standard deviation can be clearly observed. The average coefficient of variation of the test results (standard deviation divided by the average value) within each series was approximately 4%, which indicates high homogeneity of the results obtained in each group. A preliminary analysis of the strength changes shows that the average compressive strength of cement decreases with an increase in the degree of contamination. Regardless of the type of impurity, above 8% of its content in the cement composition, the strength weakening is evident. On the other hand, both cements exhibit interesting behaviour in small ranges of impurities, especially in the case of saline. Up to about 2% of its addition to the cement, average compressive strengths increased relative to pure cements.

Confirmation of the significance of the changes in the strength characteristic investigated was sought in the statistical processing of the experimental results. Analyses performed using the Tukey test (for unequal amounts of samples) allowed separating groups of homogeneous compression strength results, which are presented in Table 3. Table 4 presents cross-referenced significant differences between individual series. Values above 0.05 (black) indicate no statistically significant difference between the series being compared. The results obtained clearly show that bone cement contamination significantly affects its compressive strength. For almost every case studied, the 1% admixture significantly changed the strength, increasing it by 8.5–10% on average. Only CMW3 Gentamicin cement admixed with blood did not show any increase in compressive strength.

**Figure 5:**
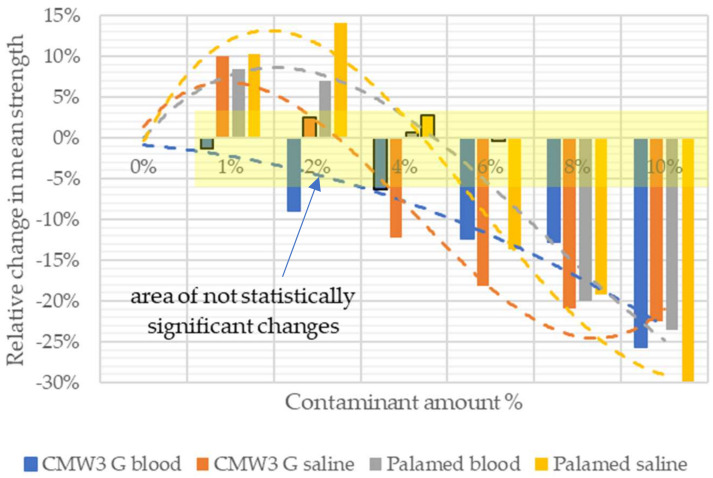
Change in average compressive strength.

A summarised comparison of the relative change in average compressive strength of the contaminated cements in relation to unmodified cement is shown in Figure 5. The results of statistical analysis have been included in the graph by framing changes not significant statistically and by defining the area of statistical non-significance.

### 3.2. Compressive Modulus of Elasticity

**Figure 6:**
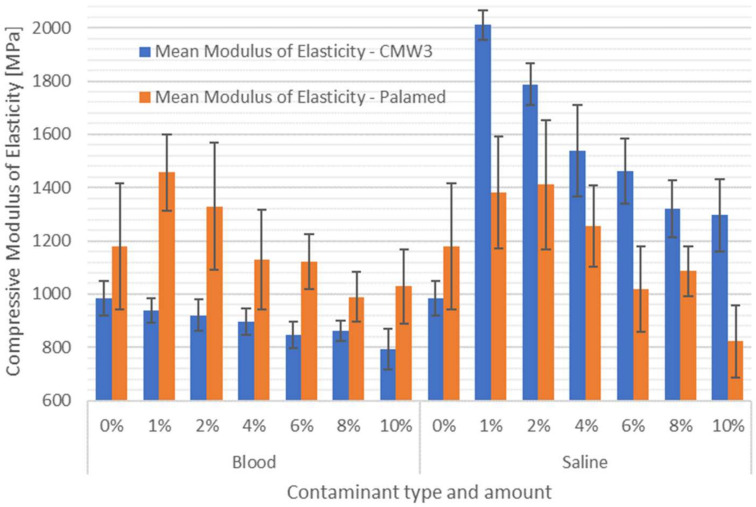
Compressive modulus of elasticity of contaminated bone cements.

A summary of the compressive modulus of elasticity is presented in Table 5 and Figure 6. The first analysis already shows clear changes in the modulus after contamination of the cements. CMW3 Gentamicin contaminated with saline solution increased its stiffness the most. The average modulus doubled already at 1% solution addition. Increasing the amount of contamination led to smaller and smaller increases in average modulus, although at 10% addition it was still about 30% more than for the uncontaminated cement. Changes of similar nature, although not so high values, were observed for Palamed cement, but the values of the distribution of the results for individual batches do not allow one to conclude at this stage whether these changes were statistically significant. The admixture of cements with blood, in the case of CMW3 Gentamicin, made the cement more and more flexible, while at 10% addition the average modulus reached about 80% of that of unadulterated cement. Larger variations were observed for Palamed, although, again, individual results with average variation are not necessarily statistically significant. However, once again similar strength behaviour of the cement was recorded, i.e., slight admixture with blood (~1%) resulted in a forced, almost jump-like increase in modulus and further admixture led to a smooth decrease in average modulus values.

The statistical analysis of the modulus of elasticity results was carried out in the same way as above for the compressive strength. Using Tukey tests, the individual results were grouped into homogeneous groups with statistically insignificant differences (Table 6). In 3 out of 4 cases of contamination, as in the case of compressive strength, the statistical significance of the change (increase) in modulus was confirmed already at 1% contamination admixture. Only the modulus of CMW3 Gentamicin cement, whose average modulus decreased from the beginning, successively with increasing contamination, showed a statistically significant decrease only at 6% contamination. The modulus of elasticity of Palamed after the initial significant increase in value, at 4% of contamination, returned to statistically insignificant change in relation to the “pure” cement and up to 8% at saline contamination, and the limit tested amount was 10% at blood contamination. Significant differences of the tested values are presented in Table 7.

**Figure 7:**
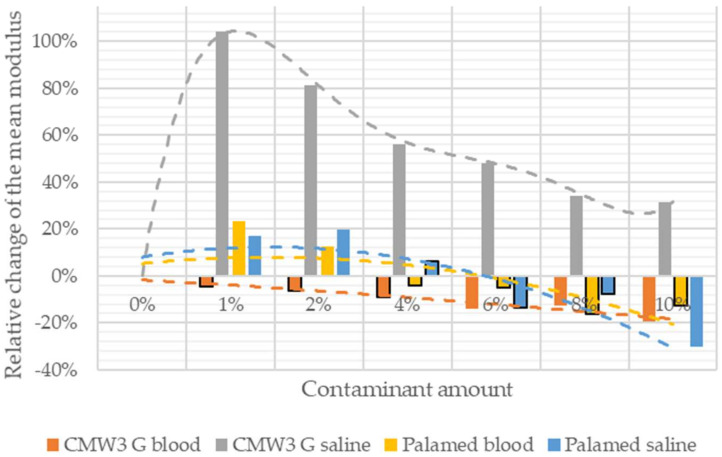
Change in mean value of the compressive modulus of elasticity.

A combined summary of the relative change in the mean value for the compressive modulus of the contaminated cements in relation to that of the unmodified cement is shown in Figure 7. The graph takes into account the results of the statistical analysis by marking statistically insignificant changes with solid frames.

Cold-cured PMMA (also known as chemically cured or self-curing PMMA) requires no thermal energy. A tertiary amine initiator such as n,n-dimethyl-ptoluidine is added to the cold-cured PMMA, which activates the benzyl peroxide, chemically generating free radicals to initiate the polymerization. In the propagation stage, the activated polymerization continues by binding monomers and is completed by shifting free electrons to the end of the chain [74,75].

Research to improve the performance of bone cements involving the admixing of different materials often leads to modification of the cement material by chemical means [34,35,76,77,78,79]. In the case of the admixtures with saline and blood described in this paper, the nature of which may be accidental and unintentional, a change in the mechanical properties of the cements was also recorded, but its reasons should not be sought in chemical interactions. The contaminants tested were chemically neutral in nature. Both saline and blood have very low chemical reactivity. The reasons for these changes should therefore be found phenomena of a physical nature. Due to the molecular polarity of the molecules, water molecules penetrate the polymer chains and act as plasticizers [80]. Thinning of the cement mass can increase the distance between cross-linking molecules and this, in turn, results in the formation of shorter polymer chains, leading to a weakening of the material. Although a significant amount of monomers will react and crosslink, the material becomes less rigid and therefore its strength decreases.

The admixture of a non-native material to an uncured cement may consequently lead to an increase in porosity of the crosslinked cement, especially if the contaminating material is a liquid that may leave the structure of the material after crosslinking the polymer. Moreover, the increase in porosity itself does not have to be regarded as a negative phenomenon as it may contribute to improving osteointegration, i.e., the biological-chemical-physical integration process that permanently links the cement to the patient’s bone [81]. This gives the fixed implant even more stabilization. An increase in porosity on a small scale can be positive from a mechanical point of view, and 1–2% admixture can be the limiting threshold. A greater increase in porosity resulting from an excessive amount of additional contaminant accidentally introduced in the cement can adversely affect mechanical performance and make the cement more susceptible to failure even when subjected to small forces.

It should be remembered that the analyzed parameters concern only the mechanical properties, and conclusions drawn from the obtained results cannot be used at this stage to construct any general recommendations. Obtaining a slight but statistically significant increase in strength with insignificant admixtures may be at the cost of deterioration of other critical properties (e.g., fatigue resistance, environmental resistance, etc.), but it provides a good basis for further research on the problem of targeted admixture to bone cements.

## 4. Conclusions

As a result of the experimental studies presented in this paper, a significant effect of the analysed impurities on the strength characteristics of the cement was demonstrated. The change in compressive strength was characterised by an initial increase, after which, after 2–4% admixture, it returned to the same values as for unmodified cement. Further increases in the number of impurities resulted in a decrease in strength compared to pure cement. Only in the case of Gentamicin CMW3 cement was such behaviour not recorded—the strength value dropped immediately (statistically significantly from about 2% of admixture). The modulus of elasticity showed a similar pattern of change with cement contamination, although there were clear differences between the two cements tested. The initial increase in modulus of the Palamed cement (blood and salt), at 2–4% contamination, changed to a decrease in modulus (increase in stiffness). CMW3 Gentamicin cement doped with blood responded (similar to compressive strength) with a decrease in modulus as the number of impurities increased (statistically significantly from 6%). Importantly, the modulus of CMW3 G cement increased approximately twice already for a minimal 1% addition of saline. The change decreased with higher amounts of impurities, but in the maximum tested range of impurities (10%) it was still statistically significant and slightly over 30% higher than unmodified cement. Thus, it is clear that proper bone preparation such as pulse lavage and drying prior to cement insertion can reduce cement degradation by contaminants and consequently reduce the percentage of cement failures after total joint replacement surgery.

## Figures and Tables

**Table 1:** Composition of examined cements.

	DePuy CMW3 GENTAMICIN	Heraeus Palamed
Powder		
	polymethyl methacrylate (PMMA)	
*initiator*	benzoyl peroxide	
*radiopaque agents*	barium sulphate	zirconium dioxide
*colorant*		E141 (chlorophyllin)
*antibiotic*	gentamicin sulphate	
**Liquid**		
	methyl methacrylate (MMA)	
*accelerator*	N,N-dimethyl-ptoluidine (DMPT)	
*stabilizer*	hydroquinone	
*colorant*		E141 (chlorophyllin)

**Table 2:** Compressive strength of examined cements in relation to the amount of contaminant.

		Blood		Saline	
	Contamination Amount	Mean Compressive Strength (MPa)	SD (MPa)	Mean Compressive Strength (MPa)	SD (MPa)
CMW3 Gentamicin	0%	75.47	1.26	75.47	1.26
	1%	74.46	1.28	82.98	1.76
	2%	68.77	3.27	77.39	2.26
	4%	70.70	5.25	66.27	2.08
	6%	66.14	1.05	61.73	1.74
	8%	65.82	1.35	59.67	1.27
	10%	56.07	3.48	58.52	2.70
Palamed	0%	63.92	4.89	63.92	4.89
	1%	69.28	1.75	70.48	2.46
	2%	68.40	3.06	72.91	3.81
	4%	64.39	2.41	65.71	1.72
	6%	63.64	1.96	55.21	2.79
	8%	51.11	1.58	51.65	2.76
	10%	48.84	2.00	44.50	2.09

**Table 3:** Homogeneous groups of results of mean compressive strength.

		Blood							Saline					
		Mean Compressive Strength (MPa)	1	2	3	4			Mean Compressive Strength (MPa)	1	2	3	4	5
CMW3 Gentamicin	0%	75.47			X		CMW3 Gentamicin	0%	75.47			X		
	1%	74.46			X			1%	82.98				X	
	2%	68.77		X				2%	77.39			X		
	4%	70.70		X	X			4%	66.27		X			
	6%	66.14		X				6%	61.73	X				
	8%	65.82		X				8%	59.67	X				
	10%	56.07	X					10%	58.52	X				
Palamed	0%	63.92		X	X		Palamed	0%	63.92			X		
	1%	69.28				X		1%	70.48				X	X
	2%	68.40			X	X		2%	72.91					X
	4%	64.39		X	X			4%	65.71			X	X	
	6%	63.64		X				6%	55.21		X			
	8%	51.11	X					8%	51.65		X			
	10%	48.84	X					10%	44.85	X				

**Table 4:** Significant differences between results of mean compressive strength.

	Contaminant	Amount of Admixture/ Mean Compressive Strength						
CMW3 Gentamicin	**Saline**	**0%**	**1%**	**2%**	**4%**	**6%**	**8%**	**10%**
		75.474	82.979	77.392	66.272	61.731	59.673	58.523
	**0%**		0.00	0.62	0.00	0.00	0.00	0.00
	**1%**	0.00		0.00	0.00	0.00	0.00	0.00
	**2%**	0.62	0.00		0.00	0.00	0.00	0.00
	**4%**	0.00	0.00	0.00		0.00	0.00	0.00
	**6%**	0.00	0.00	0.00	0.00		0.54	0.09
	**8%**	0.00	0.00	0.00	0.00	0.54		0.95
	**10%**	0.00	0.00	0.00	0.00	0.09	0.95	
	**Blood**	**0%**	**1%**	**2%**	**4%**	**6%**	**8%**	**10%**
		75.474	56.074	68.766	70.698	66.141	65.816	56.074
	**0%**		1.00	0.01	0.08	0.00	0.00	0.00
	**1%**	1.00		0.04	0.26	0.00	0.00	0.00
	**2%**	0.01	0.04		0.93	0.75	0.64	0.00
	**4%**	0.08	0.26	0.93		0.10	0.07	0.00
	**6%**	0.00	0.00	0.75	0.10		1.00	0.00
	**8%**	0.00	0.00	0.64	0.07	1.00		0.00
	**10%**	0.00	0.00	0.00	0.00	0.00	0.00	
**Palamed**	**Saline**	**0%**	**1%**	**2%**	**4%**	**6%**	**8%**	**10%**
		63.921	70.482	72.914	65.706	55.207	51.655	44.853
	**0%**		0.01	0.00	0.94	0.00	0.00	0.00
	**1%**	0.01		0.78	0.09	0.00	0.00	0.00
	**2%**	0.00	0.78		0.00	0.00	0.00	0.00
	**4%**	0.94	0.09	0.00		0.00	0.00	0.00
	**6%**	0.00	0.00	0.00	0.00		0.37	0.00
	**8%**	0.00	0.00	0.00	0.00	0.37		0.00
	**10%**	0.00	0.00	0.00	0.00	0.00	0.00	
	**Blood**	**0%**	**1%**	**2%**	**4%**	**6%**	**8%**	**10%**
		63.921	69.281	68.397	64.387	63.636	51.114	48.844
	**0%**		0.00	0.05	1.00	1.00	0.00	0.00
	**1%**	0.00		1.00	0.01	0.01	0.00	0.00
	**2%**	0.05	1.00		0.11	0.03	0.00	0.00
	**4%**	1.00	0.01	0.11		1.00	0.00	0.00
	**6%**	1.00	0.01	0.03	1.00		0.00	0.00
	**8%**	0.00	0.00	0.00	0.00	0.00		0.71
	**10%**	0.00	0.00	0.00	0.00	0.00	0.71	

Color shows the values that are statistical significantly different; Bold means delimited columns present different values/describe different objects.

**Table 5:** Compressive modulus of examined cements in relation to the amount of contaminant.

		Blood		Saline	
	Contamination Amount	Mean Compressive Modulus (MPa)	SD (MPa)	Mean Compressive Modulus (MPa)	SD (MPa)
CMW3 Gentamicin	0%	985.63	65.46	985.63	65.46
	1%	938.92	44.44	2010.83	54.60
	2%	921.44	59.03	1788.51	78.18
	4%	895.77	49.50	1539.44	171.37
	6%	848.44	49.57	1461.00	122.83
	8%	862.71	37.28	1320.94	106.20
	10%	794.87	76.02	1296.67	136.53
Palamed	0%	1179.82	237.02	1179.82	237.02
	1%	1456.79	143.96	1381.56	210.58
	2%	1328.59	238.62	1410.47	242.67
	4%	1129.35	185.60	1255.44	153.08
	6%	1122.43	103.54	1017.85	160.87
	8%	989.53	93.83	1086.30	92.30
	10%	1029.29	139.58	823.40	135.13

**Table 6:** Homogeneous groups of results of compressive modulus of elasticity.

		Blood						Saline					
		Mean Compressive Modulus (MPa)	1	2	3			Mean Compressive Modulus (MPa)	1	2	3	4	5
CMW3 Gentamicin	0%	985.63			X	CMW3 Gentamicin	0%	985.63					X
	1%	938.92		X	X		1%	2010.83				X	
	2%	921.44		X	X		2%	1788.51			X		
	4%	895.77	X	X	X		4%	1539.44		X			
	6%	848.44	X	X			6%	1461.00	X	X			
	8%	862.71	X	X			8%	1320.94	X				
	10%	794.87	X				10%	1296.67	X				
Palamed	0%	1179.82	X	X		Palamed	0%	1179.82		X	X	X	
	1%	1456.79			X		1%	1381.56			X	X	
	2%	1328.59		X	X		2%	1410.47				X	
	4%	1129.35	X	X			4%	1255.45		X	X	X	
	6%	1122.43	X	X			6%	1017.85	X	X			
	8%	989.53	X				8%	1086.30	X	X	X		
	10%	1029.29	X				10%	823.40	X				

**Table 7:** Significant differences between results of compressive modulus of elasticity.

	Contaminant	Amount of Admixture/ Mean Compressive Strength						
CMW3 Gentamicin	**Saline**	**0%**	**1%**	**2%**	**4%**	**6%**	**8%**	**10%**
		985.63	2010.8	1788.5	1539.4	1461.0	1320.9	1296.7
	**0%**		0.00	0.00	0.00	0.00	0.00	0.00
	**1%**	0.00		0.02	0.00	0.00	0.00	0.00
	**2%**	0.00	0.02		0.01	0.00	0.00	0.00
	**4%**	0.00	0.00	0.01		0.88	0.03	0.01
	**6%**	0.00	0.00	0.00	0.88		0.33	0.17
	**8%**	0.00	0.00	0.00	0.03	0.33		1.00
	**10%**	0.00	0.00	0.00	0.01	0.17	1.00	
	**Blood**	**0%**	**1%**	**2%**	**4%**	**6%**	**8%**	**10%**
		985.63	938.92	921.44	895.77	848.44	862.71	794.87
	**0%**		0.76	0.53	0.10	0.00	0.01	0.00
	**1%**	0.76		1.00	0.82	0.10	0.23	0.00
	**2%**	0.53	1.00		0.99	0.38	0.63	0.01
	**4%**	0.10	0.82	0.99		0.75	0.94	0.09
	**6%**	0.00	0.10	0.38	0.75		1.00	0.72
	**8%**	0.01	0.23	0.63	0.94	1.00		0.46
	**10%**	0.00	0.00	0.01	0.09	0.72	0.46	
Palamed	**Saline**	**0%**	**1%**	**2%**	**4%**	**6%**	**8%**	**10%**
		1179.8	1381.6	1410.5	1255.4	1017.8	1086.3	823.40
	**0%**		0.41	0.26	0.99	0.66	0.96	0.03
	**1%**	0.41		1.00	0.86	0.01	0.07	0.00
	**2%**	0.26	1.00		0.71	0.01	0.03	0.00
	**4%**	0.99	0.86	0.71		0.23	0.62	0.00
	**6%**	0.66	0.01	0.01	0.23		0.99	0.55
	**8%**	0.96	0.07	0.03	0.62	0.99		0.20
	**10%**	0.03	0.00	0.00	0.00	0.55	0.20	
	**Blood**	0%	1%	2%	4%	6%	8%	10%
		1179.8	1456.8	1328.6	1129.4	1122.4	989.53	1029.3
	**0%**		0.04	0.68	1.00	1.00	0.39	0.66
	**1%**	0.04		0.80	0.00	0.01	0.00	0.00
	**2%**	0.68	0.80		0.34	0.30	0.01	0.03
	**4%**	1.00	0.00	0.34		1.00	0.73	0.93
	**6%**	1.00	0.01	0.30	1.00		0.78	0.95
	**8%**	0.39	0.00	0.01	0.73	0.78		1.00
	**10%**	0.66	0.00	0.03	0.93	0.95	1.00	

Color shows the values that are statistical significantly different; Bold means delimited columns present different values/describe different objects.

## Data Availability

The data presented in this study are available on request from the corresponding authors.
